# Family resilience of stroke survivors within 6 months after a first-episode stroke: A longitudinal study

**DOI:** 10.3389/fpsyt.2022.968933

**Published:** 2022-10-13

**Authors:** Wei Zhang, Wanqiong Zhou, Mingming Ye, Yitian Gao, Lanshu Zhou

**Affiliations:** Department of Clinical Nursing, College of Nursing, Second Military Medical University, Shanghai, China

**Keywords:** family resilience, stroke, chronic disease, self-efficacy, coping style, social support, longitudinal study

## Abstract

**Context:**

Family resilience is frequently recognized as a powerful determinant of family adaptation in chronic disease patients; understanding the family resilience of stroke patients and its predictors could help nurses develop interventions to assist patients in maintaining healthy family functioning.

**Objective:**

This study aimed to explore the trajectory of family resilience in the 6 months following stroke onset and examine the predictors of family resilience over time.

**Methods:**

A total of 288 first-episode stroke survivors were selected from seven hospitals in China from July 2020 to March 2021. Their family resilience, social support, self-efficacy, and medical coping style were assessed at hospitalization and 1, 3, and 6 months after stroke onset. The study was performed in accordance with the STROBE guidelines.

**Results:**

The mean levels of family resilience were between 95.52 ± 11.10 and 97.68 ± 9.68 within the first 6 months after a first-episode stroke, with a significant increase 3 months after the onset. Patient self-efficacy, social support, family atmosphere, and caregiver-patient relationship (sibling) were predictors of family resilience at all four time points. Baseline predictors of family resilience at 6 months included self-efficacy of the patients, subjective support, support utilization, family atmosphere, living district, medical bill payment methods, and caregiver-patient relationship (sibling).

**Conclusion:**

Family resilience levels were low in stroke patients 6 months after the onset, and 3 months post-stroke onset was a critical period for family resilience of stroke patients. Nurses are recommended to pay particular attention to patients with low self-efficacy, perceived low support, poor utilization of available support, as well as those who are under the care of their siblings, self-pay, or live in a poor family atmosphere. Interventions aimed at improving the self-efficacy of patients and social support are potential approaches to enhance family resilience.

## Introduction

Stroke, characterized by high incidence, disability, mortality, and recurrence, typically results in physical disability and functional impairment. According to the Chinese Stroke Prevention Report, the number of stroke survivors in China has up to 12.42 million (1). Furthermore, around 75% of stroke patients were unable to live independently owing to disabilities, often in the form of hemiplegia (2). Most patients needed to live with their families, and the long-term rehabilitation process became a source of considerable physical, psychological, and economic burdens for their families. Family functioning is strongly associated with psychological recovery of stroke patients, whereas family maladjustment negatively impacts the rehabilitation outcome of patients (3). Thus, the importance of family-centered care for chronic diseases such as stroke has been emphasized by scholars (4).

The challenges faced by stroke families have been demonstrated in many studies. However, scholars began by considering family strengths recently, and the focus has shifted toward exploring family resilience, which is defined as “family strengths and harnessing family power to address the dilemmas faced by the families” (5). Families with high resilience are characterized by their ability to use internal and external family resources quickly and effectively to rebuild the endogenous strengths of the family and further promote healthy family development in the face of adversity (6). A family resilience orientation provides nurses with an opportunity to create protective factors to help foster resilience. Therefore, understanding family resilience of stroke patients and its influencing factors could aid nurses in developing interventions to further improve family resilience so that patients can cope with the crisis.

Earlier studies focused on exploring the components of family resilience (7, 8). One of the most extensively applied frameworks is the Walsh's (6) family resilience clinical practice framework, which outlines three important domains of family resilience: family belief systems, organizational processes, and communication processes, which made understanding family resilience feasible. Moreover, an increasingly important area of family care practice is identifying, strengthening, and promoting family resilience (9, 10). However, empirical studies on family resilience of stroke survivors and its predictors are limited, which is not in favor of targeted interventions for the families of post-stroke patients. Therefore, an evaluation of family resilience of stroke survivors and its predictors is crucial.

Based on the review of previous studies, family resilience is influenced by a number of factors, ranging from social and interpersonal levels to individual levels (11). At the social and interpersonal level, social support and family atmosphere seemed to be factors impacting family resilience. Social support, defined as the individual's perception or experience of support and help from social groups such as family, friends, and community (12), is believed to help families regain control and present higher family resilience (13, 14). Family atmosphere reflects the commitment, emotions, and interest of family members toward one another, thereby defining their relationship (15) and influencing their communication, problem-solving processes, and family organizational patterns. In a systematic review (16), family members' relationships and cohesion were listed as family protective factors; the indispensable role of economic resources in developing family resilience was also highlighted. However, the relationship between these factors and family resilience has not been investigated in the families of stroke patients.

At the personal level, self-efficacy and personal coping are of great interest to the researchers (16). Self-efficacy refers to the judgment of one's ability to accomplish an event (17). A study on married couples during the COVID-19 pandemic (18) determined that external stress could indirectly influence family resilience through the self-efficacy of family members. Meanwhile, Weiss et al.'s study (19) on children with autism spectrum disorders suggested that the self-efficacy of parents could predict their family resilience. In another study, the use of positive coping strategies by patients with chronic heart failure was positively correlated with family resilience (20). Moreover, individual demographic information such as caregiver's gender (21), and marital adjustment (18), education level is correlated to family resilience. However, evidence of the role of these factors in predicting the family resilience of stroke patients is lacking.

However, to the best of our knowledge, there is a lack of research on post-stroke family resilience. Considering the aforementioned points, the self-efficacy, family atmosphere, income, social support, as well as demographic and clinical characteristics of the patients were included to explore the levels and predictors of family resilience post-stroke. Since family resilience is a dynamic process (6), this study applied a longitudinal design to examine alterations in family resilience of stroke patients within 6 months after a stroke, which is a critical period for the rehabilitation of stroke patients. The relationship between the above-mentioned factors and family resilience could also be examined for a longer duration, which could be helpful for nurses to conduct timely and proper interventions for families of stroke survivors.

## Methods

### Design

The study was a longitudinal one with a convenience sample of stroke survivors.

### Participants

Stroke survivors were recruited from the neurology departments of seven tertiary hospitals in Shanghai and Shangqiu, China. All the hospitals were eligible for quick admission of stroke patients, early thrombolysis, and interventional therapy. The inclusion criteria of stroke survivors were: (a) over eighteen years old; (b) a diagnosis of first-episode stroke according to the diagnostic criteria of Chinese Society of Neurology (22, 23); (c) stable vital signs allowing participation in the survey. Exclusion criteria were: (a) presence of a psychiatric history or a mental disorder; (b) cognitive or other communication impairments; (c) other major traumatic events in the family in the past 6 months; (d) no family caregiver.

The sample size was grossly estimated according to the estimation principle that the sample size had to be 5–10 times the number of items (24). Since the maximum number of items in this study was 32, at least 160 cases had to be included. Moreover, considering a drop-out rate of 20% and a mortality rate of 5%, the sample had to comprise at least 200 cases.

### Procedures

The study was conducted in accordance with the STROBE guidelines. The purpose and process of the research were introduced to the potential participants during hospitalization, and informed consent was obtained before the study. The baseline (T0) survey was conducted after the stabilization of the vital signs of the patients; the contact information of the participants was collected at T0 for follow-ups. Follow-up surveys were conducted at 1 month (T1), 3 months (T2), and 6 months (T3) following the stroke. The follow-ups were conducted face-to-face when the patients could attend the clinics or by telephone when it was inconvenient for them to come to the clinics. When the participants were contacted *via* phone, the investigators read each item of the questionnaire one by one and accurately documented their responses. Gifts, such as towels and toothpaste, were offered to the participants face-to-face or *via* express delivery to decrease the drop-out rate. After each investigation, the questionnaires were quickly checked for missing content to ensure the questionnaire's integrity.

### Instruments

#### Family Resilience Assessment Scale

The Family Resilience Assessment Scale (FRAS) was originally developed by (25) and was based on Walsh's family resilience model. Li et al. (26) subsequently translated and modified it into the shortened Chinese version, FRAS-C, consisting of 32 items belonging to three dimensions, namely family communication and problem-solving, utilization of social resources, and positive outlook maintenance. A Likert-4 scoring method was adopted, with points ranging from 1, representing “totally disagree,” to 4, representing “totally agree.” The scale score is the sum of the scores for all the items, and the higher the score, the stronger the family resilience. The scale showed favorable reliability with a Cronbach's alpha coefficient of 0.96.

#### Social Support Rating Scale

The Social Support Rating Scale (SSRS) scale was compiled by Xiao (27) in 1986. It includes 10 items in three dimensions: objective support, subjective support, and support utilization. The total score ranges from 0 to 66, with a higher score representing a higher level of social support. According to the scale score, the level of social support could be graded as low (a score of 0 to 22), medium (a score of 23 to 44), or high (a score of 45 to 66). The scale had satisfactory reliability with a Cronbach's alpha coefficient of 0.818.

#### Self-Efficacy for Managing Chronic Disease 6-Item Scale

The Self-Efficacy for Managing Chronic Disease 6-Item Scale (SES-6), developed by the Patient Education Research Center of Stanford University, has been extensively used for evaluating the efficacy of the self-management of patients with chronic diseases (28). The Chinese version of SES-6 is a 6-item scale scoring on a Likert-10 scale, with 1 representing “no confidence” and 10 representing “full confidence.” The score of the scale is the average score of each item. The higher the score, the stronger the confidence in managing the disease. The Cronbach's alpha coefficient of the scale was determined to be 0.91.

#### Medical Coping Modes Questionnaire

The scale was compiled by Feifel et al. (29), and the Chinese version of the Medical Coping Modes Questionnaire (MCMQ) (30), a 20-item scale, is scored on a 4-point scale ranging from 1 to 4, of which eight items are reversely scored. The scale is divided into three dimensions: confrontation, avoidance, and submission. The higher the score in each dimension, the greater the likelihood that the respondent will use the coping strategy. The Cronbach's alpha coefficients of each dimension were between 0.69 and 0.76, and the retest correlation coefficients were 0.66, 0.85, and 0.69.

#### Demographic and characteristic questionnaire

The general data of the patients were obtained by a self-designed questionnaire, which included the baseline demographics of the patients and the caregivers (gender, age, religious belief, education, marriage, etc.), disease-related information (stroke type, stroke severity, etc.), and family characteristics (living region, monthly income, family atmosphere, etc.). Stroke severity was accessed with the Modified Rankin Scale (mRS), one of the most common scales for evaluating stroke outcomes in clinical practice. It consists of seven grades from 0 to 6, with 0 representing no symptoms and six representing death (31). Family atmosphere was measured with a one-item statement, “the degree of emotional cohesion between family members,” on an 11-points scale with 0 representing “very uncomfortable” and 10 representing “very warm.”

### Statistical analysis

EpiData 3.1 was used for data input and validation, while SPSS 21.0 was used for statistical analyses. The continuous data (such as age and scale scores) were expressed as mean and standard deviation, and the categorical variables were expressed as frequency and ratio. After testing normality and homogeneity of variance, *t*-test (or Mann–Whitney *U*-test) and ANOVA (or Kruskal–Wallis *H*-tests) were used for multi-group comparisons of categorical variables, while spearman correlation was used to explore the relationship between family resilience and the continuous variables. The generalized estimating equation was employed to analyze the changes of family resilience along with time. Multiple linear regression analyses were used to determine the predictors of family resilience at each time point and determine the baseline factors that could predict family resilience at 6 months. The variance inflation factor (VIF) was used to measure the collinearity among predictor variables. All the VIFs were less than five, demonstrating that the estimated models have little multi-collinearity.

### Ethical considerations

This study was conducted on the premise of passing the review of the Biomedical Research Ethics Committee of the Second Military Medical University. Written informed consent was obtained from all participants before the study, and all the information and data were kept confidential and only for the purpose of this study. The reports based on this study did not infringe upon the privacy of the participants. If the participants experienced any discomfort during the course of this investigation, the investigation would be immediately terminated. Additionally, the subjects had the right to withdraw from the study at any time.

## Results

### Characteristics of the patients

Herein, the data of 288 patients were collected at T0 (Table 1). Most of them lived in Shanghai (63.5%), were males (71.2%), and had a diagnosis of ischemic stroke (94.4%). At T1, 255 cases had completed their follow-up, while data were unavailable for 33 cases (one participant died, and 32 participants were lost to follow-up). At T2, 242 patients had completed their follow-up, while data were not available for 13 cases (one participant died, and 12 participants were lost to follow-up). At T3, 237 cases were followed-up, and five were lost to follow-up (0 participants died, five participants were lost to follow-up).

**Table 1:** Characteristics of participants at baseline, 1, 3, and 6 months after stroke onset.

**Variable**	***N*** **(%)/*****M*** ±***SD***			
	**T0 (*N* = 288)**	**T1 (*N* =255)**	**T2 (*N* = 242)**	**T3 (*N* = 237)**
**Patient**				
Male	205 (71.2)	181 (71.0)	171 (70.7)	171 (72.2)
Age	61.8 ± 12.4	61.7 ± 12.4	61.8 ± 12.3	61.7 ± 12.3
**Education**				
Junior school or below	188 (65.3)	172 (67.4)	166 (68.6)	162 (68.4)
High school	68 (23.6)	55 (21.6)	51 (21.1)	50 (21.1)
College or above	32 (11.1)	28 (11.0)	25 (10.3)	25 (10.5)
Ischemic stroke	272 (94.4)	241 (94.5)	230 (95.0)	225 (94.9)
Rankin score	2.34 ± 1.35	2.35 ± 1.34	2.33 ± 1.34	2.31 ± 1.34
With religious belief	16 (5.6)	15 (5.9)	15 (6.2)	15 (6.3)
**Marriage status**				
Unmarried	14 (4.7)	11 (4.3)	11 (4.5)	11 (4.6)
Married	259 (87.8)	230 (90.2)	218 (90.1)	214 (90.3)
Divorce	4 (1.4)	3 (1.2)	3 (1.2)	3 (1.3)
Widowed	11 (3.7)	11 (4.3)	10 (4.1)	9 (3.1)
**Work status**				
Unemployed	85 (29.5)	75 (29.4)	72 (29.8)	69 (29.1)
Leave/retire	145 (50.3)	128 (50.2)	122 (50.4)	121 (51.1)
On-the-job	58 (20.1)	52 (20.4)	48 (19.8)	47 (19.8)
**Caregiver**				
Male	106 (35.9)	94 (36.9)	89 (36.8)	86 (36.3)
Age	49.1 ± 14.1	48.7 ± 13.9	49.2 ± 13.4	49.2 ± 13.3
**Relationship with patient**				
Spouse	130 (44.1)	113 (44.3)	110 (45.5)	109 (46.0)
Children	129 (43.7)	115 (45.1)	106 (43.8)	102 (43.0)
Parent	16 (5.4)	15 (5.9)	14 (5.8)	14 (5.9)
Sibling	13 (4.4)	12 (4.7)	12 (5.0)	12 (5.1)
With religious belief	29 (9.8)	26 (10.2)	25 (10.3)	25 (10.5)
**Education**				
Junior school or below	157 (53.2)	143 (56.1)	140 (57.8)	137 (57.8)
High school	64 (21.7)	55 (21.6)	50 (20.7)	48 (20.3)
Colleges or above	67 (22.7)	57 (22.4)	52 (21.5)	52 (21.9)
**Marriage status**				
Unmarried	25 (8.5)	23 (9.0)	16 (6.6)	15 (6.3)
Married	255 (86.4)	226 (88.6)	220 (90.9)	216 (91.1)
Divorce	7 (2.4)	5 (2.0)	5 (2.1)	5 (2.1)
Widowed	1 (0.3)	1 (0.4)	1 (0.4)	1 (0.4)
**Work status**				
Unemployed	76 (25.8)	69 (27.1)	63 (26.0)	62 (26.2)
Leave/retire	97 (32.9)	82 (32.2)	81 (33.5)	80 (33.8)
On-the-job	115 (39.0)	104 (40.8)	98 (40.5)	95 (40.1)
With chronic disease	65 (22.0)	56 (22.1)	55 (22.7)	53 (22.4)
**Family**				
Living region				
Shanghai	183 (63.5)	161 (63.1)	155 (64.0)	152 (64.1)
Shangqiu	105 (36.5)	94 (36.9)	87 (36.0)	85 (35.9)
**Monthly income (RMB)**				
< 1,000	9 (3.1)	6 (2.4)	6 (2.5)	6 (2.5)
1,000–3,000	97 (32.9)	88 (34.5)	86 (35.5)	82 (34.6)
3,000–5,000	130 (44.1)	115 (45.1)	106 (43.8)	105 (44.3)
5,000–10,000	43 (14.6)	39 (15.3)	37 (15.3)	37 (15.6)
>10,000	9 (3.1)	7 (2.7)	7 (2.9)	7 (3.0)
**Payment style**				
Self-pay	35 (12.2)	32 (12.5)	30 (12.4)	29 (12.2)
Medical insurance	163 (56.6)	143 (56.1)	134 (55.4)	132 (55.7)
New rural cooperative medical insurance	90 (31.3)	80 (31.4)	78 (32.2)	76 (32.1)
Family atmosphere	8.05 ± 1.555	8.05 ± 1.532	8.03 ± 1.548	8.06 ± 1.538

N, number; M, mean; SD, standard deviation.

### Descriptive data

The descriptions of the key variables are presented in Table 2. The average FRAS-C score at baseline was 95.52 (SD = 11.10), with an upward trend for the following three time points, reaching a mean score of 97.68 (SD = 9.68) at T3.

**Table 2:** Descriptions of key variables at four time points.

**Variable**	***M*** **(*****SD*****)**			
	**T0 (*N* = 288)**	**T1 (*N* = 255)**	**T2 (*N* = 242)**	**T3 (*N* = 237)**
**Family resilience**	95.52 ± 11.10	96.19 ± 9.87	97.28 ± 9.58	97.68 ± 9.68
FCPS	69.47 ± 8.09	69.58 ± 7.21	70.88 ± 6.99	71.39 ± 7.18
USR	7.84 ± 1.76	8.13 ± 1.51	7.95 ± 1.54	7.85 ± 1.56
MPO	18.21 ± 2.33	18.47 ± 2.69	18.45 ± 2.00	18.43 ± 1.93
**Medical coping**
Confrontation	17.50 ± 2.61	18.10 ± 2.24	18.28 ± 2.12	19.38 ± 1.90
Avoidance	16.91 ± 1.92	17.36 ± 1.88	14.33 ± 2.47	17.25 ± 1.86
Resignation	12.03 ± 1.780	12.38 ± 1.80	12.76 ± 1.61	12.86 ± 2.06
Self-efficacy	7.27 ± 1.48	7.55 ± 1.50	8.14 ± 1.34	8.24 ± 1.38
Social support	34.73 ± 7.51	35.39 ± 6.83	35.31 ± 6.54	35.71 ± 6.70
Objective support	7.99 ± 2.54	8.34 ± 2.38	8.49 ± 2.23	8.77 ± 2.35
Subjective support	20.89 ± 4.57	21.17 ± 4.14	20.73 ± 4.13	20.95 ± 4.11
Support utilization	5.86 ± 2.40	5.88 ± 2.42	6.09 ± 2.24	6.05 ± 2.24

FCPS, family communication and problem solving; USR, Utilizing social resources; MPO, Maintaining a positive outlook; M, mean; SD, standard deviation.

### Changes of family resilience within the first 6 months after stroke

Generalized estimating equation showed an increase in FRAS-C along with time (χ^2^ = 28.414, *p* < 0.001). Compared with T0, there was a significant increase in the FRAS-C score at T2 and T3 (*p* < 0.05), while no significant differences were observed at T1 (Table 3).

**Table 3:** Results of generalized estimating equation of the family resilience (*N* = 237).

	***B***	**SE**	**95% CI of Exp (** ***B*** **)**		**Wald χ^2^**	***p*-Value^b^**	**Total Wald χ^2^**	***df***	***p*-Value for model**
			**Lower**	**Upper**					
T3	2.158	0.500	1.179	3.138	8.641	< 0.001	31.379	3	< 0.001
T2	1.760	0.388	0.999	2.521	20.568	< 0.001			
T1	0.667	0.420	−0.155	1.49	2.531	0.112			
T0	0^a^								

β', Standardized Coefficients; T0, during hospitalization; T1, 1 month after onset; T2, 3 months after onset; T3, 6 months after onset; CI, confidence interval.

^a^Set zero because this parameter is redundant.

^b^P < 0.017 was considered statistically significant.

### Predictors of family resilience at baseline, 1, 3, and 6 months after stroke

Exploratory univariate analyses exposed that factors significantly correlated with FRAS-C scores at T0, T1, T2, and T3 were patients' work status, self-efficacy, social support, family atmosphere, and caregiver's education. Patient's educational attainment, Rankin score, caregiver's gender and relationship with the patient, and monthly family income were significantly correlated with FRAS-C scores at T0, T1, and T2. In contrast, patients' age, marital status, caregivers' age, religious belief, work status, medical expense payment methods, and living region were related to FRAS-C scores at one or two time points.

The results of multiple linear regression models revealed that predictors of family resilience were: self-efficacy, subjective support, family atmosphere, resignation, divorce (patient), self-pay, caregiver's educational attainment (junior high school or below), and caregiver-patient relationship (sibling) at T0 (*R*^2^ = 0.37); self-efficacy, subjective support, family atmosphere, and caregiver-patient relationship (sibling) at T1 (*R*^2^ = 0.27); self-efficacy, subjective support, family atmosphere, and caregiver-patient relationship (sibling) at T2 (*R*^2^ = 0.30); and self-efficacy, availability, family atmosphere, region, self-pay, caregiver-patient relationship (sibling) at T3 (*R*^2^ = 0.27; Table 4).

**Table 4:** Multiple linear regressions for predictors of family resilience at four time points.

	**Predictors**	**β^′^**	***t***	***p*-Value**	**95% CI of the difference**		***R* ^2^**	**Adjusted *R*^2^**
					**Lower**	**Upper**		
T0 (*N* = 288)	Constant		10.712**	< 0.001	41.325	59.932	0.383	0.365
	Subjective support-T0	0.265	4.762**	< 0.001	0.378	0.910		
	Self-efficacy-T0	0.186	3.547**	< 0.001	0.621	2.171		
	Family atmosphere	0.205	3.684**	< 0.001	0.681	2.243		
	Resignation-T0	0.162	3.349*	0.001	0.411	1.583		
	Self-pay	−0.111	−2.285*	0.023	−6.982	−0.519		
	Divorce (patient)	−0.140	−2.956*	0.003	−12.476	−2.501		
	Caregiver-patient relationship (sibling)	−0.150	−3.110*	0.002	−23.120	−5.198		
	Caregiver education (junior high school or below)	−0.122	−2.517*	0.012	−4.833	−0.591		
T1 (*N* = 255)	Constant		17.090**	< 0.001	55.903	70.466	0.281	0.270
	Subjective support-T1	0.250	4.158**	< 0.001	0.314	0.878		
	Self-efficacy-T1	0.242	4.195**	< 0.001	0.842	2.331		
	Family atmosphere	0.172	2.888**	0.004	0.352	1.863		
	Caregiver-patient relationship (sibling)	−0.230	−4.243**	< 0.001	−15.641	−5.724		
T2 (*N* = 242)	Constant		15.861**	< 0.001	54.931	70.513	0.310	0.298
	Family atmosphere	0.288	4.748**	< 0.001	1.041	2.517		
	Subjective support-T2	0.210	3.446**	< 0.001	0.209	0.766		
	Self-efficacy-T2	0.203	3.574**	< 0.001	0.653	2.256		
	Caregiver-patient relationship (sibling)	−0.193	−3.556**	< 0.001	−13.222	−3.795		
T3 (*N* = 237)	Constant		12.968**	< 0.001	53.562	72.754	0.294	0.273
	Self-efficacy-T3	0.228	3.933**	< 0.001	0.760	2.287		
	Support utilization-T3	0.275	4.850**	< 0.001	0.706	1.672		
	Family atmosphere	0.189	2.993**	0.003	1.300	6.308		
	Living region	0.231	3.621**	< 0.001	0.663	2.244		
	Self-pay	−0.165	−2.901**	0.004	−8.182	−1.563		
	Caregiver-patient relationship (sibling)	−0.214	−3.757**	< 0.001	−14.408	−4.495		

β', Standardized Coefficients; T0, during hospitalization; T1, 1 month after onset; T2, 3 months after onset; T3, 6 months after onset; CI, confidence interval.

*Correlation is significant at 0.05 level.

**Correlation is significant at 0.01 level.

### Baseline predictors of family resilience at 6 months

Univariate analyses demonstrated that the baseline variables associated with FRAS-C at T3 were living region (*Z* = 5.00, *p* < 0.001), medical expense payment methods (*H* = 14.78, *p* = 0.001), patient marital status (*H* = 9.76, *p* = 0.021), patient work status (*H* = 7.66, *p* = 0.022), self-efficacy (*r* = 0.21, *p* = 0.001), subjective support (*r* = 0.14, *p* = 0.035), availability (*r* = 0.21, *p* = 0.001), caregiver educational attainment (*H* = 7.87, *p* = 0.049), caregiver work status (*H* = 6.01, *p* = 0.050), and family atmosphere (*r* = 0.50, *p* < 0.001) (Table 5). Multiple linear regression analyses uncovered that self-efficacy, support utilization, living region, subjective support, family atmosphere, self-pay method for medical expense, and caregiver-patient relationship (sibling) were predictors of FRAS at 6 months.

**Table 5:** Multiple linear regression for baseline predictors of family resilience at 6 months (*N* = 237).

**Predictors**	**β^′^**	***t***	***p*-Value**	**95% CI of the difference**		***R* ^2^**	**Adjusted *R*^2^**
				**Lower**	**Upper**		
Constant		12.040**	< 0.001	51.575	71.761	0.293	0.268
Self-efficacy-T0	0.165	2.731**	0.007	0.316	1.955		
Support utilization -T0	0.234	3.858**	< 0.001	0.449	1.385		
Living region	0.256	3.846**	< 0.001	2.511	7.786		
Subjective support-T0	0.170	2.456*	0.015	0.073	0.661		
Family atmosphere	0.174	2.570*	0.011	0.255	1.933		
Self-pay	−0.160	−2.795**	0.006	−8.039	−1.391		
Caregiver-patient relationship (sibling)	−0.204	−3.544**	< 0.001	−13.971	−3.986		

β', Standardized Coefficients; T0, during hospitalization; T1, 1 month after onset; T2, 3 months after onset; T3, 6 months after onset; CI, confidence interval.

*Correlation is significant at 0.05 level.

**Correlation is significant at 0.01 level.

## Discussion

This study aimed to investigate the trajectory and predictors of family resilience of stroke patients within 6 months following the stroke. The family resilience was low early after the stroke, and there was a significant increase 3 months after the stroke. Self-efficacy of patients, social support, family living region, family atmosphere, self-funding of medical expenses, and being cared for by siblings were independent predictors of family resilience at 6 months.

The mean level of family resilience of stroke survivors was between 95.52 ± 11.10 and 97.68 ± 9.68 within the first 6 months after stroke, lower than that of cancer patients (*M* = 150.81, SD = 13.56) (32), which could be attributed to the early disease stage in this study and that “the stroke onset leads to a sudden and unexpected change in the family roles, resulting in the weakening of the family strength” (33). Results also demonstrated that the family resilience score was increased within the first 6 months, with a significant increase 3 months after onset. However, contrary to the findings of this study, a study conducted by Chen et al. (34) on adolescents/young adults with a parent diagnosed with cancer showed a lower family resilience over time, with a significant decrease at 6 months. The variations might be due to multiple distinct diseases, which need to be further validated in future studies.

In this study, social support was strongly correlated with family resilience of stroke patients, with subjective support predicting family resilience within the first 3 months and support utilization predicting that at 6 months. This result is consistent with the findings of Chen et al. (32) concerning families of cancer patients and the study of Liu and Xiong (35) on families bereft of their only child. The findings herein further signal that the perceived support of the patients was more important for family resilience within 3 months, and the support utilization of stroke patients became important 6 months after stroke. Moreover, baseline social utilization could predict family resilience at 6 months. Therefore, as a resource of social support, nurses could play a role by providing psychological support to increase the subjective support of stroke patients (36) and by helping the patients realize the support available to promote support utilization further and enhance family resilience.

In line with findings of a previous study (17, 18), self-efficacy was demonstrated to be one of the strongest predictors of family resilience, implying that a high self-efficacy of patients in managing stroke might increase family resilience and assist their families in coping with changes brought on by the stroke. Therefore, promoting the confidence of patients and the conviction in their ability to attain their recovery goals could increase not only the resilience of the patients (37) but also that of their families. Group interventions have been proven to outperform individual interventions in enhancing the self-efficacy of patients (38); hence, nurses organizing group education or patient communication and sharing sessions, as well as providing digital follow-ups might be feasible approaches to improving patient self-efficacy following discharge (39), and also potentially enhancing family resilience.

To the best of our knowledge, this is the first study to explore the relationship between the coping styles of stroke patients and family resilience. The results signal that only resignation was a predictor of family resilience at baseline. However, our current findings do not provide a clear explanation for the occurrence of the aforementioned phenomenon, and further quantitative or qualitative studies are warranted to corroborate this finding.

At the family level, family atmosphere was a positive predictor of family resilience at all four time points. A positive family atmosphere could be a therapeutic environment for patients (40). In contrast, out-of-pocket medical expenses were a negative predictor of family resilience at hospitalization and played a role 6 months after stroke onset. This can be attributed to the fact that hospitalization after a stroke involves significant expenses, which can be an unexpected financial setback for the families of stroke patients (41), making them less resilient during hospitalization. Besides, stroke has a long recovery period, imposing a long-term burden on families (42), especially in terms of finance, due to the possibility of stroke recurrence in the later stages of recovery (43). This could explain why out-of-pocket medical expenses had a detrimental impact on family resilience 6 months following the stroke.

Moreover, patients cared for by their siblings rather than their spouses or children tended to experience lower family resilience within the first 6 months following a stroke. In the current study, 44.1% of the patients were cared for by their spouses and 43.1% by their children, consistent with the observations of a previous study (44), indicating that stroke patients were primarily cared for by their spouses and children. However, special attention should be paid to those families with stroke patients cared for by their siblings because that they would be less resilient.

Interestingly, the living region was also a predictor of family resilience 6 months after onset. For instance, living in Shangqiu positively predicted the family resilience of stroke patients, probably because families in Shangqiu were more likely to be extended families (45, 46) and were under less living strain compared to those in Shanghai (47). Besides, the findings demonstrated that divorced patients and patients with a less-educated caregiver were more likely to experience low family resilience during hospitalization. Previous research also reported similar results. Marital status has been found to influence resilience in families, and couple relationships are resilient to meet changing needs (48, 49). Educated caregivers may have an advantage in terms of acquiring the knowledge and skills necessitated to look after stroke patients (50), resulting in a higher family resilience to help families through the crisis.

The current study also found that self-efficacy of patients, subjective support, support utilization of the patients, being cared for by their siblings, family living region, family atmosphere, and ways to cover medical expenses during hospitalization were independent predictors of family resilience at 6 months. The predictive effect of these baseline factors suggests that nurses should pay special attention to the families of patients with low self-efficacy, poor ability in support utilization and family atmosphere, perceiving less social support, and being cared for by their siblings and at their own expense during hospitalization. However, although a longitudinal study, this study used a correlational design, so it is not possible to figure out the causal role of variables such as self-efficacy in family resilience. In the future, experimental studies are needed to investigate the causal effects of the potential influencing factors.

### Limitations

There were several limitations to this study that need to be taken into account. First, the patients were enrolled from two urban areas of two cities, limiting the generalizability of the outcome to other cities and rural areas. Second, the influencing factors were predominantly studied from the standpoint of patients, accounting for only 26.8%−36.5% of variances in family resilience of stroke patients, implying that there might be other confounding factors. Third, stroke recovery is a long-term process, but family resilience was investigated within only the first 6 months post-stroke. Further research, including additional factors for a longer follow-up period, is required to gain a deeper understanding of post-stroke family resilience. Finally, although this was a longitudinal study, it was a quantitative one, and the daily life of family members and their interactions could not be assessed. In the future, a combination of quantitative and qualitative studies may enable a comprehensive assessment and understanding of family resilience.

## Conclusion

Understanding family resilience in stroke patients and its influencing factors will help nurses develop interventions to assist patients in maintaining functioning families. This study discovered that although there was a significant increase in family resilience 3 months after a stroke, the resilience of families within the first 6 months post-stroke was low compared to families with members suffering from other health conditions. During hospitalization, nurses are recommended to pay particular attention to patients with low self-efficacy, perceived low support, poor utilization of available support, as well as those who are under the care of their siblings, self-pay, or live in a poor family atmosphere; since they might suffer from a low level of family resilience 6 months after stroke onset. Interventions aimed at improving the self-efficacy of patients and social support are potential approaches to enhance family resilience.

## Data availability statement

The raw data supporting the conclusions of this article will be made available by the authors, without undue reservation.

## Ethics statement

The studies involving human participants were reviewed and approved by the Institutional Review Board of Second Military Medical University (NMUMREC-2021-017). The patients/participants provided their written informed consent to participate in this study.

## Author contributions

WZha: conceptualization, methodology, interpretation of data, writing the original draft, review and editing, and approval of the final version. WZho: interpretation of data, writing the original draft, and approval of the final version. MY: data acquisition and analysis, review and editing, and approval of the final version. LZ: conceptualization, methodology, review and editing, and approval of the final version. All authors contributed to the article and approved the submitted version.

## Funding

The study was funded by the youth program of the National Natural Science Foundation of China (grant number 71904197), the major project of the National Social Science Foundation of China (grant number 21&ZD188), and the Sailing project of Second Military Medical University. The funders had no role in study design, data collection and analysis, decision to publish, or preparation of the manuscript.

## Conflict of interest

The authors declare that the research was conducted in the absence of any commercial or financial relationships that could be construed as a potential conflict of interest.

## Publisher's note

All claims expressed in this article are solely those of the authors and do not necessarily represent those of their affiliated organizations, or those of the publisher, the editors and the reviewers. Any product that may be evaluated in this article, or claim that may be made by its manufacturer, is not guaranteed or endorsed by the publisher.
