# Supplementary material for: Family resilience of stroke survivors within 6 months after a first-episode stroke: A longitudinal study
Source: Front Psychiatry. 2022 Oct 13;13:968933. doi: 10.3389/fpsyt.2022.968933 (PMC9606654; doi:10.3389/fpsyt.2022.968933)
Supplement: Supplementary file 1 [file Table_1.DOCX]

Supplementary Table. Comparisons of FRAS-C scores at four time points

| **Variable** | **T0(*N*=288)** | | | **T1(*N*=255)** | | | **T2(*N*=242)** | | | **T3(*N*=237)** | | |
| --- | --- | --- | --- | --- | --- | --- | --- | --- | --- | --- | --- | --- |
|  | M±SD | Statistics | p | M±SD | Statistics | p | M±SD | Statistics | p | M±SD | Statistics | p |
| **PATIENT** | | | | | | | | | | | | |
| **Gender** |  |  |  |  |  |  |  |  |  |  |  |  |
| male | 96.09±11.95 | -2.132(z) | .033* | 96.34±10.48 | -.479(z) | .632 | 97.98±10.53 | -2.037(z) | .038* | 97.60±10.10 | .79(z) | .428 |
| female | 94.12±8.54 |  |  | 95.81±8.26 |  |  | 95.59±6.53 |  |  | 97.88±8.58 |  |  |
| **Age** | / | .055(r) | .350 | / | .013(r) | .833 | / | .001(r) | .991 | / | -.06(r) | .335 |
| **Education** |  |  |  |  |  |  |  |  |  |  |  |  |
| junior school or below | 93.70±9.25 | 30.068(h) | <.001** | 94.61±8.88 | 17.585(h) | <.001** | 95.92±8.35 | 15.620 | <.001** | 97.21±9.10 | 1.60(h) | .449 |
| high school | 97.22±13.24 |  |  | 98.78±11.12 |  |  | 98.76±10.34 |  |  | 98.22±10.82 |  |  |
| colleges or above | 102.63±12.90 |  |  | 100.79±10.85 |  |  | 103.28±12.82 |  |  | 99.64±11.00 |  |  |
| **Stroke type** |  |  |  |  |  |  |  |  |  |  |  |  |
| ischemic | 95.42±11.19 | .845(z) | .398 | 96.19±10.03 | .233(z) | .816 | 97.35±9.77 | .017(z) | .986 | 97.77±9.89 | -.470(z) | .639 |
| hemorrhagic | 97.19±9.59 |  |  | 96.14±6.83 |  |  | 95.92±4.46 |  |  | 96.00±4.31 |  |  |
| **Rankin score** | / | -.206(r) | <.001** | / | -.148(r) | .018* | / | -.240(r) | <.001** | / | -.049(r) | .456 |
| **Family history** |  |  |  |  |  |  |  |  |  |  |  |  |
| No | 95.45±10.70 | .840(z) | .401 | 96.37±9.13 | -1.104(z) | .270 | 97.49±9.15 | .110(z) | .912 | 97.78±8.94 | -1.403(z) | .161 |
| Yes | 95.79±12.52 |  |  | 95.53±12.27 |  |  | 96.54±11.00 |  |  | 97.35±11.96 |  |  |
| **Religious belief** |  |  |  |  |  |  |  |  |  |  |  |  |
| No | 95.53±11.08 | -.009(z) | .993 | 96.22±9.94 | .076(z) | .940 | 97.28±9.48 | .340(z) | .734 | 97.93±9.78 | -1.029(z) | .303 |
| Yes | 95.37±11.83 |  |  | 95.73±9.02 |  |  | 97.27±11.40 |  |  | 94.00±7.48 |  |  |
| **Marriage** |  |  |  |  |  |  |  |  |  |  |  |  |
| Unmarried | 93.86±9.66 | 18.197(h) | <.001** | 92.27±11.12 | 17.161(h) | .001** | 95.09±10.05 | 16.589(h) | .001** | 95.64±10.22 | 9.759(h) | .021* |
| Married | 96.08±11.14 |  |  | 96.92±9.45 |  |  | 97.89±9.34 |  |  | 98.08±9.69 |  |  |
| Divorce | 76.75±9.29 |  |  | 76.33±10.69 |  |  | 74.00±9.17 |  |  | 83.00±4.36 |  |  |
| Widowed | 91.18±3.37 |  |  | 90.18±9.90 |  |  | 93.30±2.91 |  |  | 95.44±5.88 |  |  |
| **Work status** |  |  |  |  |  |  |  |  |  |  |  |  |
| Unemployed | 91.79±6.16 | 28.568(h) | <.001** | 94.15±5.81 | 7.294(h) | .026* | 94.42±4.55 | 15.521(h) | <.001** | 97.75±5.95 | 7.658(h) | .022* |
| Leave/retire | 96.52±12.77 |  |  | 96.60±11.46 |  |  | 97.84±10.99 |  |  | 97.14±11.49 |  |  |
| On-the-job | 98.48±10.95 |  |  | 98.12±9.93 |  |  | 100.15±10.31 |  |  | 98.96±9.04 |  |  |
| **Monthly income** |  |  |  |  |  |  |  |  |  |  |  |  |
| ＜1000 | 85.00±20.04 | 33.121(h) | <.001** | 91.33±6.31 | 9.606(h) | .048* | 94.33±6.65 | 15.482(h) | .004** | 92.50±5.86 | 2.798(h) | .592 |
| 1000-3000 | 92.52±9.07 |  |  | 94.38±9.23 |  |  | 95.24±8.28 |  |  | 97.24±8.12 |  |  |
| 3000-5000 | 96.59±10.40 |  |  | 96.66±9.93 |  |  | 97.85±9.67 |  |  | 98.17±10.52 |  |  |
| 5000-10000 | 100.28±11.48 |  |  | 98.77±9.85 |  |  | 100.32±10.39 |  |  | 98.14±9.94 |  |  |
| ＞10000 | 100.22±14.00 |  |  | 101.00±14.81 |  |  | 100.14±15.89 |  |  | 97.43±14.91 |  |  |
| **Family atmosphere** | / | .459(r) | <.001** | / | .290(r) | <.001** | / | .422(r) | <.001** | / | .496(r) | <.001** |
| **CAREGIVER** | | | | | | | | | | | | |
| **Gender** |  |  |  |  |  |  |  |  |  |  |  |  |
| Male | 93.67±8.62 | 2.319(z) | .020* | 93.93±9.108 | 3.086(z) | .002** | 94.39±7.22 | 3.116(z) | .002** | 96.00±7.93 | 1.469(z) | .142 |
| Female | 96.60±12.21 |  |  | 97.51±10.08 |  |  | 98.96±10.37 |  |  | 98.64±10.459 |  |  |
| **Age** | / | 0.144(r) | .015* | / | 0.100(r) | .110 | / | .081(r) | .208 | / | -.108(r) | .098 |
| **Relationship with patient** |  |  |  |  |  |  |  |  |  |  |  |  |
| Spouse | 97.75±11.68 | 9.641(h) | .022* | 98.38±10.68 | 11.946(h) | .008** | 99.35±10.63 | 9.132(h) | .028* | 98.94±10.78 | 7.521(h) | .057 |
| Children | 94.07±10.37 |  |  | 95.21±7.93 |  |  | 96.02±7.66 |  |  | 97.31±8.12 |  |  |
| Parent | 94.62±9.72 |  |  | 94.00±12.01 |  |  | 96.36±9.89 |  |  | 96.93±9.97 |  |  |
| Sibling | 88.77±9.45 |  |  | 87.67±10.47 |  |  | 90.58±10.40 |  |  | 90.25±8.35 |  |  |
| **Religious belief** |  |  |  |  |  |  |  |  |  |  |  |  |
| No | 95.90±11.12 | -1.579(z) | .114 | 96.52±9.89 | -1.907(z) | .057 | 97.73±9.52 | -2.205(z) | .027* | 98.12±9.88 | -1.799(z) | .072 |
| Yes | 92.17±10.47 |  |  | 93.27±9.34 |  |  | 93.36±9.33 |  |  | 93.92±6.84 |  |  |
| **Education** |  |  |  |  |  |  |  |  |  |  |  |  |
| Junior high school or below | 93.70±9.25 | 16.377(h) | <.001** | 94.88±8.95 | 6.174(h) | .046* | 95.94±8.25 | 7.324(h) | .026* | 96.58±8.64 | 6.185(h) | .045* |
| High school | 97.22±13.24 |  |  | 97.15±10.22 |  |  | 99.28±10.42 |  |  | 99.17±9.94 |  |  |
| Colleges or above | 102.63±12.90 |  |  | 98.54±11.28 |  |  | 98.98±11.49 |  |  | 99.19±11.67 |  |  |
| **Marriage** |  |  |  |  |  |  |  |  |  |  |  |  |
| Unmarried | 96.52±11.97 | 2.640(h) | .451 | 95.78±10.40 | 2.434(h) | .487 | 99.62±12.44 | 1.449(h) | .694 | 100.93±12.79 | 2.810(h) | .422 |
| Married | 95.52±11.15 |  |  | 96.25±9.92 |  |  | 97.14±9.45 |  |  | 97.55±9.54 |  |  |
| Divorced/widowed | 91.29±5.16 |  |  | 93.60±4.72 |  |  | 95.00±5.15 |  |  | 94.20±3.56 |  |  |
| **Work status** |  |  |  |  |  |  |  |  |  |  |  |  |
| Unemployed | 92.80±7.22 | 14.819(h) | .001** | 95.03±7.01 | 1.787(h) | .409 | 95.4±6.51 | 5.794(h) | .055 | 97.55±7.082 | 6.011(h) | .050 |
| Leave/retire | 97.19±14.41 |  |  | 97.01±12.82 |  |  | 98.46±12.03 |  |  | 97.16±12.061 |  |  |
| On-the-job | 95.91±9.67 |  |  | 96.31±8.78 |  |  | 97.47±8.85 |  |  | 98.20±8.950 |  |  |
| **With chronic disease** |  |  |  |  |  |  |  |  |  |  |  |  |
| No | 94.70±10.78 | 1.694(z) | .090* | 95.57±9.24 | 1.173(z) | .241 | 96.92±9.21 | .936(z) | .349 | 97.21±9.111 | .589(z) | .556 |
| Yes | 98.32±11.79 |  |  | 98.38±11.67 |  |  | 98.51±10.73 |  |  | 99.32±11.396 |  |  |
| **Region** |  |  |  |  |  |  |  |  |  |  |  |  |
| Shanghai | 97.21±13.04 | -4.129(z) | <.001** | 96.91±11.71 | -.798(z) | .425 | 98.37±11.28 | -2.145(z) | .032* | 96.92±11.45 | 5.000(z) | <.001** |
| Shangqiu | 92.57±5.34 |  |  | 94.95±5.27 |  |  | 95.33±4.84 |  |  | 99.04±4.98 |  |  |
| **Payment** |  |  |  |  |  |  |  |  |  |  |  |  |
| self-pay | 93.03±8.31 | 8.662(h) | .013* | 93.88±8.97 | 2.821(h) | .244 | 94.43±6.89 | 4.015(h) | .134 | 93.38±6.52 | 14.777(h) | .001** |
| medical insurance | 97.06±13.23 |  |  | 96.79±11.52 |  |  | 98.51±11.47 |  |  | 98.10±11.56 |  |  |
| NRCMI | 93.71±6.49 |  |  | 96.04±6.34 |  |  | 96.27±5.93 |  |  | 98.59±6.13 |  |  |
| **Family atmosphere** | / | .278(r) | <.001** | / | .224(r) | <.001** | / | .260(r) | <.001** | / | .142(r) | .029* |
| **Medical Coping** |  |  |  |  |  |  |  |  |  |  |  |  |
| Confrontation | / | .227(r) | <.001** | / | .216(r) | .001** | / | .092(r) | .154 | / | -.095(r) | .145 |
| Avoidance | / | .132(r) | .025** | / | .079(r) | .211 | / | .149(r) | .021* | / | .053(r) | .419 |
| Resignation | / | .161(r) | .006** | / | .088(r) | .159 | / | .080(r) | .213 | / | .276(r) | <.001** |
| **Self-efficacy** | / | .391(r) | <.001** | / | .377(r) | <.001** | / | .382(r) | <.001** | / | .362(r) | <.001** |
| **Social support** | / | .514(r) | <.001** | / | .409(r) | <.001** | / | .449(r) | <.001** | / | .200(r) | .002** |
| Objective support | / | .359(r) | <.001** | / | .268(r) | <.001** | / | .368(r) | <.001** | / | .135(r) | .038* |
| Subjective support | / | .483(r) | <.001** | / | .368(r) | <.001** | / | .409(r) | <.001** | / | .095(r) | .147 |
| support utilization | / | .294(r) | <.001** | / | .254(r) | <.001** | / | .205(r) | <.001** | / | .252(r) | <.001** |

Note: N, number; M, mean; SD, standard deviation; r, Spearman correlation analysis; Z, Mann-Whitney U-test; H, Kruskal-Wallis H-test.

* Correlation is significant at 0.05 level; **Correlation is significant at 0.01 level.

NRCMI: New rural cooperative medical insurance
